# Translucency and color match with a shade guide of esthetic brackets with the aid of a spectroradiometer

**DOI:** 10.1590/2177-6709.21.2.081-087.oar

**Published:** 2016

**Authors:** Yong-Keun Lee, Yu Bin

**Affiliations:** 1 Director, Institute for Clinical Performance of Biomaterials (ICPB) and ETN Dental Clinic, Seoul, Korea.; 2 Associate Professor, Chinese Academy of Sciences, State Key Laboratory of Multiphase Complex Systems, Institute of Process Engineering, Beijing, China

**Keywords:** Orthodontic bracket, Color compatibility, Translucency, Esthetic performance, Spectroradiometer.

## Abstract

**Objective:**

Since the color of esthetic brackets should match that of teeth, the aims of this study were to determine the color and translucency of esthetic brackets by means of the clinically relevant use of a spectroradiometer, and to compare the color of brackets with that of a commercial shade guide.

**Methods:**

The color of central and tie-wing regions of four plastic and four ceramic brackets was measured according to the CIE L*a*b* color scale over white and black backgrounds. Brackets were classified into five groups based on their composition. The color of Vitapan Classical Shade Guide tabs was also measured. Translucency parameter (TP) and contrast ratio (CR) were calculated to determine translucency.

**Results:**

Color differences between brackets and the shade guide tabs were 10.4 - 34.5 ∆E*_ab_ units. TP and CR values for the central region were 16.4 - 27.7 and 0.38 - 0.58, whereas for the tie-wings they were 24.0 - 39.9 and 0.25 - 0.45, respectively. The color coordinates, TP and CR values were significantly influenced by bracket composition and brand (*p* < 0.05).

**Conclusions:**

Esthetic brackets investigated herein showed unacceptable color differences (∆E*_ab_ > 5.5) compared with the shade guide tabs. Differences in the translucency of brackets by brand were within the visually perceptible range (∆CR > 0.07). Therefore, brackets showing the best matching performance for each case should be selected considering esthetic and functional demands.

## RESUMO

**Objetivo:**

considerando-se que a cor dos braquetes estéticos deve corresponder à cor dos dentes, os objetivos do presente estudo foram determinar a cor e a translucidez de braquetes estéticos, por meio do uso clinicamente relevante de um espectroradiômetro, e comparar a cor desses braquetes com a cor de uma escala comercial de cores.

**Métodos:**

a cor das regiões central e da aleta de quatro braquetes plásticos e quatro braquetes cerâmicos foi medida segundo o sistema de cores CIE L*a*b*, sobre fundos branco e preto. Os braquetes foram classificados em cinco grupos, de acordo com a sua composição. A cor das peças da escala de cores Vitapan Classical^(r)^ também foi medida. O parâmetro de translucidez (PT) e a razão de contraste (RC) foram calculados para se determinar a translucidez.

**Resultados:**

as diferenças de cor entre os braquetes e as peças da escala de cores variaram de 10,4 a 34,5 unidades ∆E*_ab_. Os valores de PT e RC, respectivamente, variaram de 16,4 a 27,7 e de 0,38 a 0,58 na região central; já para a região das aletas, esses valores variaram de 24,0 a 39,9 e de 0,25 a 0,45. As coordenadas de cor e os valores de PT e RC foram significativamente influenciados pela marca e composição dos braquetes (*p* < 0,05).

**Conclusões:**

os braquetes estéticos investigados nesse estudo demonstraram diferenças de cor inaceitáveis (∆E*_ab_ > 5,5), quando comparados às peças da escala de cores. As diferenças de translucidez entre as marcas de braquetes estavam dentro do limite visualmente perceptível (∆RC > 0,07). Portanto, ao selecionar os braquetes com a melhor correspondência de cor, deve-se levar em consideração as exigências estéticas e funcionais de cada caso.

## INTRODUCTION

The increasing need for esthetic orthodontic treatment has led to the development of acceptable esthetic appliances, such as those with plastic and ceramic brackets.^1^ ^,^ ^2^ ^,^ ^3^ Initially, plastic brackets were made of acrylic resin and polycarbonate;^2^ ^,^ ^4^ later on, polyurethane brackets reinforced with ceramic or glass fillers were introduced.^1^ ^,^ ^2^ These improved brackets showed significantly better clinical performance;^1^ ^,^ ^2^ however, even improved plastic brackets showed clinically unacceptable color stability in the long-term.^5^ Ceramic brackets provide better color stability than plastic ones.^1^ ^,^ ^2^ ^,^ ^6^ Although the term "ceramic" encompasses a variety of compounds, most ceramic brackets are composed of either polycrystalline or monocrystalline aluminium oxide.^7^ ^,^ ^8^

Color and translucency of esthetic brackets have several clinical considerations.^1^ ^,^ ^9^ ^,^ ^10^ Firstly, bracket color should either match the color of teeth or bracket should be translucent enough to allow the underlying color to shine through. Secondly, since light-curing orthodontic adhesives are light-cured through brackets, polymerization of adhesives is influenced by the translucency of brackets.^11^

Quantitative color match performance of esthetic material is usually determined by the Commission Internationale de L'Eclairage (CIE) L*a*b* color difference (∆E*_ab_) value. Physical meanings of color difference values have been provided,^12^ ^,^ ^13^ ^,^ ^14^ and 2.6 ∆E*_ab_ units was considered as a clinically perceptible threshold, while 5.5 ∆E*_ab_ units was considered as an acceptable threshold, based on spectroradiometer readings.^12^ Translucency of dental material has been usually determined with the aid of parameters, such as light transmittance, translucency parameter (TP) and/or contrast ratio (CR).^11^ ^,^ ^13^ ^,^ ^15^ The higher the TP value or the lower the CR value, the higher the translucency. TP and CR values correspond directly to common visual assessments of translucency.^13^ As to the relationship between instrumental translucency measurement and subjective visual assessment, the mean translucency perception threshold (∆CR) was 0.07, while 50% of participants could perceive 0.06 ∆CR between specimens.^16^

A few studies directly measured the color and translucency of esthetic brackets,^9^ ^,^ ^10^ ^,^ ^17^ mainly because contact type spectrophotometers are designed to measure the color of a big object flat surface.^9^ Nowadays, noncontact type spectroradiometer has been introduced in Dentistry.^18^ ^,^ ^19^ Spectroradiometers were used to measure the color of ceramics,^18^ ^,^ ^19^ teeth,^20^ shade guide tabs^21^ and esthetic brackets,^22^ as well as the translucency of resin composites.^23^ Color and translucency values of irregularly shaped objects, measured by means of a spectroradiometer, have several strengths. A spectroradiometer provides clinically relevant values because this measurement can eliminate the edge-loss effect,^20^ ^-^ ^23^ closely simulate clinical viewing conditions,^18^ ^,^ ^24^ ^,^ ^25^ and also provide optical values of a specified area, such as wings or body of a bracket. According to a spectroradiometer-based bracket color study,^22^ color blending of brackets placed on less chromatic or lighter shade guide tabs was better than that on more chromatic or darker tabs; however, color and translucency ranges of esthetic brackets by composition or brand were not provided.

The aims of this study were to determine the color and translucency of esthetic brackets with a noncontact type spectroradiometer and to compare the color of brackets with that of a commercial shade guide. The null hypothesis assumed in the present study was that the color and translucency of esthetic brackets would not be different by the composition and brand of brackets based on noncontact type color measurements.

## MATERIAL AND METHODS

### Material

Four plastic and four ceramic bracket brands were investigated (n = 5 for each brand; Table 1). All brackets were Roth prescription for maxillary central incisors with 0.018-in slot. Two plastic (ES-P and SP-P) and one ceramic (CL-C) bracket had a metal-lined archwire slot. According to the composition, the brackets were classified into five groups.

**Table 1:** Brackets investigated.

Material	Composition	Code	Brand name	Batch number	Manufacturer
Plastic	Hybrid polymer	ES-P	Esther II	197-101R	Tomy Orthodontics, Tokyo, Japan.
	Glass-reinforced plastic	IM-P	Image	IM-11-45	Gestenco International, Gothenburg, Sweden.
	Ceramic-reinforced plastic	SI-P	Silkon Plus	002922M	American Orthodontics, Sheboygan, WI, USA.
		SP-P	Spirit MB	494-0110	Ormco, Orange, CA, USA.
Ceramic	Polycrystalline	CL-C	Clarity	6400-601	3M Unitek, Monrovia, CA, USA.
		CR-C	Crystalline V	165-101R	Tomy Orthodontics, Tokyo, Japan.
		SI-C	Signature III	KQ9042	RMO, Denver, CO, USA.
	Monocrystalline	IN-C	Inspire ICE	443-0110	Ormco, Orange, CA, USA.

* The size of all brackets was 0.018-in, Roth for UR1.

### Methods

**Figure 1:**
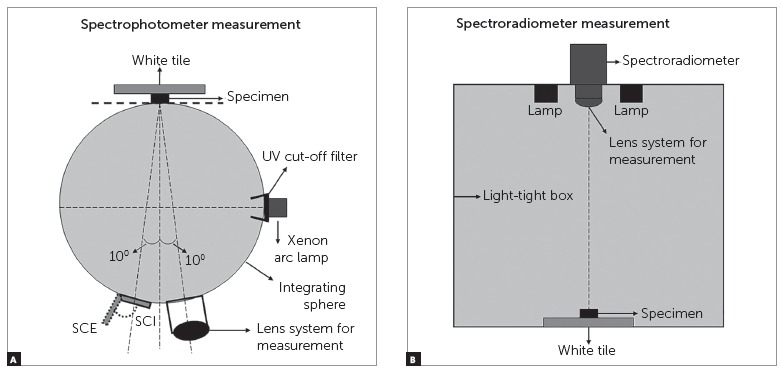
Color measurement geometries for the spectrophotometer (A) and spectroradiometer (B).^18^ ^,^ ^19^

**Figure 2:**
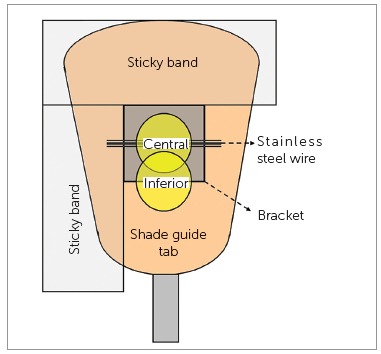
Experimental set up.^22^

A spectroradiometer equipped with Macro-Spectar MS-75 lens (PR 670, Photo Research, Chatsworth, CA, USA) was fixed vertically in a light-tight box (Color Sense II, Sungjin Hi-Tech, Gunpo, Korea), 450 mm above the specimen. The measuring geometry was d/0 (i.e. diffuse illumination/0° viewing angle). Two F20T12/65 6500K lamps (standard illuminant D65 simulator; GretagMacbeth, New Windsor, NY, USA) were fixed to the inner top surface of the light-tight box parallely (Fig 1).^18^ ^,^ ^19^ The color of a 3-mm diameter area in the central region of the bracket and 1-mm diameter region in three out of four tie-wings was measured according to the CIE L*a*b* color scale over a white (CIE L* = 94.4, a* = -0.1 and b* = -0.6) and a black background (CIE L* = 5.3, a* = -0.4 and b* = -1.4). Measurements were repeated three times for the central region and once for each one of the three tie-wings. The color of the tie-wing with the color-coded ID dot was not measured. 1-mm area was measured in tie-wings because of the limited size of this region. To simulate clinical situations, a stainless steel wire (0.016 x 0.022-in; Ormco, Glendora, CA, USA) was placed into the slot of each bracket during the color measurement procedure (Fig 2).^22^ The color of a 3-mm diameter area in the central region of 16 Vitapan Classical Shade Guide (VITA Zahnfabrik, Bad Sackingen, Germany) tabs was also measured.

In the CIE L*a*b* color space, CIE L* is a measure of lightness. The CIE a* value is a measure of redness or greenness and CIE b* is a measure of yellowness or blueness. Chroma was calculated^26^ as C*_ab_ = (a*^2^ + b*^2^)^1/2^. TP was calculated as: TP = [(L_W_* - L_B_*)^2^ + (a_W_*- a_B_*)^2^ + (b_W_*- b_B_*)^2^ ]^1/2^, in which the subscripts W and B refer to color coordinates over white and black backgrounds, respectively.^22^ CR was calculated as the ratio of bracket reflectance when it was placed on the black background (Y_b_) to that of the same specimen when it was placed over the white background (Y_w_): CR = Y_b_/Y_w_. The CIE L*a*b* color difference between brackets and shade guide tabs was calculated with the equation: ∆E*_ab_ = [(∆L*)^2^ + (∆a*)^2^ + (∆b*)^2^]^1/2^, based on the color coordinates at the central region of brackets measured over the white background.

### Statistical analysis

To determine the differences in color coordinates and translucency, two-way analysis of variance (ANOVA), was used according to bracket composition and brand (SPSS 16.0, Chicago, IL, USA). The means, representing bracket composition and brand, were compared by means of Scheffe's multiple comparison test (α = 0.05). Pearson correlations between TP and CR, and between TP values of the central region and the tie-wings, were determined.

## RESULTS

CIE L* and C*_ab_, as well as CIE a* and b* values in the central region of brackets over the white background are given in Table 2. The ranges for CIE L*, a*, b* and C*_ab_ were 75.8 to 98.3, -1.4 to 0.3, 2.6 to 8.0, and 2.6 to 8.1, respectively. All color coordinates were influenced by bracket composition and brand (*p* < 0.05). Based on Scheffe's multiple comparison test, the monocrystalline ceramic bracket (IN-C) showed the highest L* value. Ceramic-reinforced plastic brackets (SI-P) and ceramic policrystaline brackets (CL-C) showed the lowest lightness, whereas ceramic-reinforced plastic brackets (SI-P and SP-P) showed the highest chroma (C*_ab_) and polycrystalline ceramic bracket (CL-C) the lowest chroma.

**Table 2:** CIE L*, C*_ab_, a* and b* values in the central region of brackets over a white background.

Code	CIE L*	CIE C*_ab_	CIE a*	CIE b*
ES-P	80.7 (1.1)^c^	4.6 (0.5)^C^	-0.2 (0.1)^de^	4.6 (0.5)^C^
IM-P	82.0 (0.5)^cd^	5.0 (0.4)^CD^	-0.5 (0.2)^c^	5.0 (0.4)^CD^
SI-P	77.6 (0.6)^ab^	8.1 (0.2)^E^	-1.4 (0.1)^a^	8.0 (0.2)^E^
SP-P	78.0 (0.5)^b^	5.8 (0.3)^D^	-1.0 (0.1)^b^	5.7 (0.3)^D^
CL-C	75.8 (0.5)^a^	2.6 (0.1)^A^	0.1 (0.1)^ef^	2.6 (0.1)^A^
CR-C	83.1 (0.7)^d^	4.8 (0.4)^C^	-0.5 (0.1)^c^	4.8 (0.4)^C^
IN-C	98.3 (0.7)^f^	2.7 (0.3)^AB^	-0.4 (0.2)^cd^	2.7 (0.4)^AB^
SI-C	86.7 (1.0)^e^	3.6 (0.3)^B^	0.3 (0.1)f	3.5 (0.3)^B^

Standard deviations are within parentheses. There were no significant differences by brand marked with the same lowercase (CIE L* and CIE a*) or uppercase (C*_ab_ and CIE b*) letters (*p* > 0.05).

**Figure 3:**
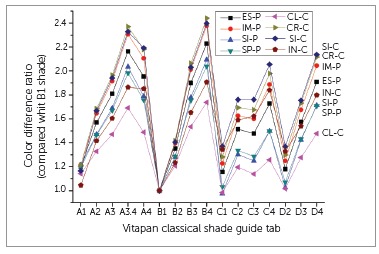
Ratios for the color differences between brackets and shade tabs as compared with the B1 shade tab. All color difference values were based on the color of the central region of brackets over a white background.

Color differences (∆E*_ab_) between brackets and Vitapan Classical Shade Guide tabs were within the range of 10.4 to 34.5 ∆E*_ab_ units. CL-C bracket showed the smallest color differences with the shade tabs, while SI-C bracket showed the largest color differences (Fig 3). B1 tab always showed the smallest color difference with brackets. Ratios for color differences between brackets and the shade tabs, compared with that of the B1 tab, are shown in Figure 3. More chromatic tabs always showed higher color difference ratios as compared to less chromatic tabs in the same hue series, such as A, B, C and D.

**Figure 4:**
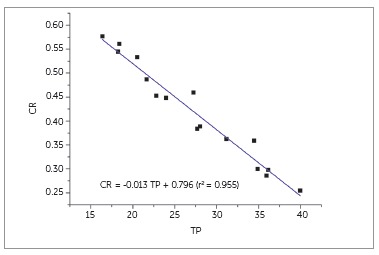
Correlation between TP and CR values of brackets.

TP and CR values of brackets are given in Table 3 for the central region and the tie-wings. TP and CR values were 16.4 to 27.7 and 0.38 to 0.58 for the central region, and 24.0 to 39.9 and 0.25 to 0.45 for the tie-wings, respectively. TP values of the central region and tie-wings showed significant correlation (coefficient of determination; r^2^ = 0.48, *p* < 0.01). TP and CR values were significantly influenced by bracket composition and brand based on two-way ANOVA (*p* < 0.05). Based on Scheffe's tests, the following order was obtained for TP of the central region (*p* < 0.05): polycrystalline ceramic (mean: 18.4); ceramic-reinforced plastic (20.5); ceramic-reinforced plastic and hybrid polymer (21.7); monocrystalline ceramic (27.2) and glass-reinforced plastic (27.7). The correlation between TP and CR values is shown in Figure 4. CR values were negatively correlated with TP values (r^2^ = 0.955, *p* < 0.05).

**Table 3:** TP and CR values at central and tie-wing regions of brackets.

Code	TP		CR	
	Central	Tie-wings	Central	Tie-wings
ES-P	21.7 (2.1)*^c^	34.9 (2.9)^cd^	0.49 (0.04)^B^	0.30 (0.03)^AB^
IM-P	27.7 (0.9)^d^	39.9 (4.0)^d^	0.38 (0.02)^A^	0.25 (0.03)^A^
SI-P	22.8 (1.0)^c^	35.9 (1.8)^cd^	0.45 (0.02)^B^	0.29 (0.02)^AB^
SP-P	18.2 (1.5)^ab^	28.0 (0.8)^ab^	0.54 (0.03)^CD^	0.39 (0.01)^CD^
CL-C	16.4 (1.2)^a^	24.0 (1.4)^a^	0.58 (0.03)^D^	0.45 (0.02)^D^
CR-C	18.4 (0.6)^ab^	36.1 (3.1)^cd^	0.56 (0.01)^CD^	0.30 (0.04)^AB^
SI-C	20.5 (0.6)^bc^	31.2 (1.4)^bc^	0.53 (0.01)^C^	0.36 (0.02)^BC^
IN-C	27.2 (1.2)^d^	34.5 (1.1)^d^	0.46 (0.02)^B^	0.36 (0.01)^BC^

* Standard deviations are within parentheses.

There were no significant differences by brand marked with the same lowercase (TP) or uppercase (CR) letters (*p* > 0.05).

## DISCUSSION

The null hypotheses of this study was rejected because all color coordinates and translucency of the investigated brackets were influenced by bracket composition and brand (*p* < 0.05). This *in vitro* study also found that the color of esthetic brackets was unacceptably different from that of any tab of Vitapan Classical Shade Guide (∆E*_ab_>5.5).^12^ Ideally, esthetic brackets should match the color of natural teeth. Since the color coordinates of natural teeth vary by race, sex and age,^27^ shade guide tabs, which are regarded to represent the color ranges of natural teeth, were used in this study. However, the color of brackets was measured over a white background with light reflectance of nearly 100%, whereas shade tabs themselves had their original color; therefore, the measuring conditions for brackets and shade tabs were not identical. Therefore, color difference ratios to that of B1 tab were compared in the present study (Fig 3). All brackets showed similar trends in color difference ratios with the shade tabs.

Recently, acceptability and perceptibility thresholds for color differences in Dentistry were reviewed and concluded that more than half of studies defined perceptibility threshold as ∆E*_ab_= 1, and one third of studies referred to ∆E*_ab_= 3.7 as the threshold at which 50% of observers accepted the color difference.^14^ However, threshold values of 2.6 and 5.5, respectively, were used in the present study because these values were based on spectroradiometer measurements.^12^ It is clear that color difference values are influenced by the measuring instrument.^20^

In a previous study, color of esthetic brackets was measured with a spectrophotometer over a zero calibration cylinder, and it was reported that all color coordinates were influenced by the brand of bracket.^10^ In the present study, the central region of brackets was measured over a white background and color coordinates were found to be significantly influenced by bracket brand, even in the same composition group (Table 2). Significantly higher L* values, in the present study, were attributed to the difference in backgrounds used in two studies. The white background had light reflectance of nearly 100%, whereas the zero calibration cylinder had light reflectance of nearly 0.^10^ Among the brackets investigated, monocrystalline ceramic bracket (IN-C) showed the highest lightness and the lowest chroma, which would make it suitable for use on bleached teeth.

Because the color of esthetic brackets and natural teeth is hard to be matched, translucency is an important optical attribute of esthetic brackets. Highly translucent brackets would allow the underlying tooth color to shine through them. In the present study, it was found that translucency was significantly influenced by bracket composition and brand. As to the discrepancies in color and translucency of brackets made of the same material, differences in thickness and geometry of brackets, especially in the tie-wings, might be the main reason.

Based on the TP value of the central region, glass-reinforced plastic and monocrystalline ceramic brackets were significantly more translucent than the other brackets, which might be attributed to their different microstructures (Table 3). Transparency of glass fillers in the glass-reinforced plastic bracket and lack of grain boundaries in the monocrystalline ceramic bracket could account for the high translucency of these brackets.^1^ These results are in agreement with a previous study^10^ which measured diffuse light transmittance value with a spectrophotometer to describe the translucency of esthetic brackets. Translucency at the tie-wing of the glass-reinforced plastic bracket was significantly greater than other bracket types, and the hybrid polymer plastic bracket was more translucent than the polycrystalline ceramic brackets (Table 3). Due to different thicknesses and geometries of the brackets, a weak, but statistically significant correlation was observed between TP values of the central region and the tie-wings (r^2^ = 0.48).

Based on the results of the present study, CR and TP values were significantly and negatively correlated, which was in agreement with previous studies based on other types of material.^15^ ^,^ ^28^ As the coefficient of determination between CR and TP was 0.955, TP and CR values may be used interchangeably. However, the clinical implications of these findings require further study. Since the mean CR value of eight brackets was 0.50, and the range was 0.38 to 0.58 (Table 3), the translucency difference was perceptible in many pairs of brackets compared, based on the translucency perception threshold (∆CR) of 0.07.^16^

The strengths of this study included that the color and translucency of esthetic brackets were directly determined by means of a noncontact color measuring instrument. Additionally, this study proved quantitatively that the color of esthetic brackets and natural teeth (shade guide tabs in this study) rarely match, hence it highlighted the importance of translucency of esthetic brackets.

There were also several weaknesses. Firstly, the shade guide tabs as substitutes for natural teeth might have not represented the true color range of natural teeth. Secondly, only limited brands of esthetic brackets were examined. Thirdly, color and translucency of esthetic brackets might change with use, but only new brackets were investigated in the present study. Lastly, although the numerical data of the present study indicated that color differences between brackets and the shade guide tabs were higher than the acceptable limit, there have been several limitations in experimental methods of the present study. Therefore, clinical color matching performance might be better than that extrapolated from the *in vitro* numerical values.

The clinical implications of the present study were that orthodontists have to consider not only the color and translucency of brackets when choosing the most esthetic appliance, but also the shade of patient's teeth. In addition to color and translucency, the shape and thickness of brackets varied by bracket brand, which would also influence the esthetic performance.

## CONCLUSIONS

Color coordinates and translucency of brackets investigated with a spectroradiometer were significantly influenced by composition and brand. Translucency difference by bracket brand was perceptible (∆CR > 0.07). All investigated brackets showed unacceptable color differences compared with all of Vitapan Classical Shade Guide tabs, based on *in vitro* experimental results. Therefore, brackets with the best matching performance for each case should be selected considering esthetic and functional demands.
